# Phase I study of the anti-TIGIT antibody tiragolumab in combination with atezolizumab in Japanese patients with advanced or metastatic solid tumors

**DOI:** 10.1007/s00280-023-04627-3

**Published:** 2024-01-11

**Authors:** Noboru Yamamoto, Takafumi Koyama, Jun Sato, Tatsuya Yoshida, Kazuki Sudo, Satoru Iwasa, Shunsuke Kondo, Kan Yonemori, Atsuko Kawasaki, Kyoko Satake, Shoyo Shibata, Toshio Shimizu

**Affiliations:** 1 Department of Experimental Therapeutics, National Cancer Center Hospital, Tsukiji 5-1-1, Chuo-Ku, Tokyo, 104–0045 Japan; 2 Chugai Pharmaceutical Co., Ltd, 1-1 Nihonbashi-Muromachi 2-Chome Chuo-Ku, Tokyo, 103–8324 Japan

**Keywords:** Atezolizumab, Tiragolumab, Phase I study, Japanese patients, Solid tumors, TIGIT

## Abstract

**Purpose:**

Tiragolumab is a monoclonal antibody that binds to the inhibitory immune checkpoint TIGIT (T-cell immunoreceptor with Ig and ITIM domains). In early phase clinical trials, tiragolumab in combination with the programmed death-ligand 1-inhibitor atezolizumab was well tolerated and has demonstrated preliminary anti-tumor activity in patients with advanced/metastatic solid tumors. We report the results of a phase I study of tiragolumab plus atezolizumab in Japanese patients (jRCT2080224926).

**Methods:**

Japanese patients ≥ 20 years old received tiragolumab (600 mg) and atezolizumab (1200 mg) intravenously every 21 days until unacceptable toxicity or disease progression. Primary endpoints were safety and pharmacokinetic (PK) parameters of tiragolumab plus atezolizumab. Secondary endpoints were anti-tumor activity.

**Results:**

Three patients were enrolled with diagnoses of non-small cell lung cancer, pancreatic cancer, and cholangiocarcinoma. No dose-limiting toxicities were observed. Two patients experienced treatment-related adverse events (AEs) of any grade. There were no grade ≥ 3 AEs, serious AEs, AEs leading to discontinuation, modification or withdrawal of any study drug, or AEs leading to death. At cycle 1, mean PK parameters of tiragolumab were as follows: C_max_ 217 μg/mL; C_min_ 54.9 μg/mL; area under the concentration–time curve from 0 to the last measurable concentration, 2000 μg·day/mL; t_1/2_, 17.6 days. Best overall response was stable disease in two patients.

**Conclusion:**

Tiragolumab plus atezolizumab was well tolerated in Japanese patients with advanced/metastatic solid tumors, and no differences in tiragolumab PK characteristics were noted between Japanese patients enrolled in this study, and non-Japanese patients enrolled in a global phase Ia/Ib study. These results may support the inclusion of Japanese patients in ongoing global phase III clinical trials.

**Trial registration number:**

jRCT2080224926.

**Supplementary Information:**

The online version contains supplementary material available at 10.1007/s00280-023-04627-3.

## Introduction

The development of cancer immunotherapy such as immune checkpoint inhibitors (ICIs) has revolutionized the treatment of patients with several advanced/metastatic solid tumors. Several ICIs are now approved by the US Food and Drug Administration, including cytotoxic T-lymphocyte-associated protein-4, programmed death-1 (PD-1), and programmed death-ligand 1 (PD-L1) inhibitors, with indications across 19 different tumor types and two tissue-agnostic conditions [1]. However, despite the availability of predictive biomarkers for response, only a small proportion of patients achieve a durable anti-tumor response to ICI monotherapy [1], most likely due to development of resistance or tumor heterogeneity [2, 3]. Thus, novel therapies or combination regimens are needed to induce more durable responses.

TIGIT, the T-cell immunoreceptor with Ig and ITIM domains, is a novel inhibitory immune checkpoint expressed on the surface of activated T cells and natural killer cells that binds with high affinity to CD155 (the poliovirus receptor [PVR]) [4–6]. TIGIT is overexpressed in multiple human cancers and strongly correlated with CD8 and PD-1 expression, particularly in tumor-infiltrating lymphocytes (TILs) [5, 6]. Simultaneous inhibition of the TIGIT/PVR and PD-L1/PD-1 pathways improved anti-tumor activity compared with blockade of only one pathway in mouse tumor models [5], and increased in vitro proliferation, cytokine production, and anti-tumor function of CD8 + TILs from patients with hepatocellular carcinoma or melanoma [7, 8]. Antibody blockade of TIGIT is currently being explored in a number of clinical studies, primarily in combination with anti-PD-L1/PD-1 antibodies. Tiragolumab is a fully human IgG1/kappa anti-TIGIT monoclonal antibody with an intact Fc region that prevents the binding of TIGIT to PVR that is well tolerated and has demonstrated promising anti-tumor activity in combination with the PD-L1-inhibitor atezolizumab in phase I and II clinical trials [9–12]. In the phase II CITYSCAPE study (NCT03563716), tiragolumab combined with atezolizumab resulted in a clinically meaningful improvement in objective response rate, progression-free survival (PFS), and overall survival in the intent-to-treat population, driven by the PD-L1-high subgroup, compared with placebo combined with atezolizumab in the first-line setting for patients with metastatic non-small cell lung cancer (NSCLC) [11, 12].

The global, multicenter, open-label phase Ia/Ib dose-escalation and dose-expansion study (GO30103; NCT02794571) investigated the safety, pharmacokinetic (PK), and preliminary anti-tumor activity of tiragolumab as a single agent and in combination with the PD-L1-inhibitor atezolizumab in patients with advanced/metastatic solid tumors [13]. In that study tiragolumab was well tolerated when administered as a single agent or in combination with atezolizumab; there were no dose-limiting toxicities (DLTs), and the recommended phase II dose identified was 600 mg given by intravenous (IV) infusion every 21 days. Most treatment-related adverse events (AEs) and immune-mediated AEs were grade 1/2 in severity with 4% of patients experiencing Grade 3/4 treatment-related AEs in both phase Ia (n = 1) and phase Ib (n = 2). The exposure of tiragolumab increased with increasing dose, and its PK characteristics were not altered when administered in combination with atezolizumab. Tiragolumab in combination with atezolizumab showed promising anti-tumor activity, mainly in the subset of patients with cancer immunotherapy-naïve PD-L1-positive solid tumors. We conducted a phase I study (JO41789) to confirm the recommended phase II dose (600 mg every 21 days) and to evaluate the safety, tolerability, and PK of tiragolumab in combination with atezolizumab (1200 mg every 21 days) in Japanese patients with advanced or metastatic solid tumors.

## Materials and methods

### Study design and patients

This was an open-label phase I study (jRCT2080224926; JO41789) conducted at the National Cancer Central Hospital, Tokyo, Japan, to investigate the safety, tolerability, and PK of tiragolumab in combination with atezolizumab in Japanese patients with advanced or metastatic solid tumors for whom standard treatment was ineffective, inadequate, or not yet established.

Eligible patients were aged ≥ 20 years, with histologically or cytologically confirmed solid tumors, an Eastern Cooperative Oncology Group performance status (ECOG PS) of 0 or 1, lesions evaluable on imaging by Response Evaluation Criteria in Solid Tumors (RECIST) v1.1, and ≥ 12 weeks life expectancy. Patients were excluded if, prior to enrollment, they had: undergone major surgery within 4 weeks; received chemotherapy (including targeted agents and antibodies) or radiotherapy within 3 weeks; received cancer immunotherapy (including ICIs and co-stimulating agonists) within 6 weeks; received a blood transfusion, hematopoietic factor products, endocrine therapy (except hormonal replacement therapy, oral contraceptives, and gonadotropin-releasing hormone agonist/antagonist) or immunosuppressive therapy within 2 weeks; or live attenuated vaccines or other study drugs within 4 weeks. Other key exclusion criteria included: patients with a history of hypersensitivity to the excipients of tiragolumab or atezolizumab; patients with persistent AEs from prior therapy of grade ≥ 2; patients who had previously received cancer immunotherapy who experienced immune-related AEs leading to discontinuation of therapy, immune-related AEs of grade ≥ 3, or unresolved immune-related AEs of grade < 2; patients with concomitant or previous autoimmune disease; and patients with central nervous system or leptomeningeal metastases with symptoms or requiring treatment.

The study protocol was reviewed and approved by the institutional review board or independent ethics committee at the study center, and the study was conducted according to the ethical principles of the Declaration of Helsinki. All patients provided written informed consent.

### Treatment

Based on the results of the phase Ia/Ib GO30103 study [13], the dose of tiragolumab was recommended to be 600 mg given by IV infusion on day 1 of each 21-day cycle, followed by atezolizumab given by IV infusion at a dose of 1200 mg on day 1 of each 21-day cycle. DLT was assessed during cycle 1. No dose reductions of study treatment were permitted, and dose delays were permitted. If administration of tiragolumab and atezolizumab were delayed for more than 42 days, then discontinuation was considered. If atezolizumab was discontinued, tiragolumab should also be discontinued. However, if tiragolumab was discontinued, single-agent atezolizumab could be continued as long as clinical benefit was perceived by the investigator. Patients were treated until disease progression (PD), death, unacceptable toxicity, or patient/investigator decision to discontinue.

### Study objectives and assessments

The primary objective was to evaluate the safety (including determination of DLTs and AEs), tolerability, and PK parameters of tiragolumab plus atezolizumab combination therapy. The secondary objective was to assess the preliminary anti-tumor activity of the combination regimen, including response rate, disease control rate, best overall response, duration of response, and PFS. Exploratory objectives were to evaluate biomarkers and the immunogenicity of tiragolumab plus atezolizumab, including development of anti-drug antibodies (ADAs). Exploratory biomarker analysis will not be reported in this paper.

The DLT assessment period was 21 days (cycle 1) (see Online Resource). DLTs and AEs were graded according to the National Cancer Institute Common Terminology Criteria for Adverse Events v5.0 and encoded using the Medical Dictionary for Regulatory Activities v22.1. Blood samples for determination of tiragolumab and atezolizumab PK were collected on day 1 of cycles 1, 2, 3, 4, and 8, and throughout cycle 1 (days 2, 8, and 15; tiragolumab only). Tiragolumab and atezolizumab serum concentrations were measured using a validated enzyme-linked immunosorbent assay. To determine the serum concentration of atezolizumab, a validated ELISA with a lower limit of quantification of 60 ng/mL was used. To determine the serum concentrations of tiragolumab, a validated ELISA with a lower limit of quantification of 25 ng/mL was used. Estimated PK parameters included minimum plasma concentration (C_min_), maximum plasma concentration (C_max_), area under the concentration–time curve to the last measurable concentration (AUC_last_), and elimination half-life (t½). Serum samples for determination of ADAs were collected on day 1 of cycles 1, 2, 3, 4, and 8, and assessed using a validated immunoassay. Tumor assessments were performed on day 1 of cycles 3, 5, and 7, and on day 1 of every 4 cycles from cycle 9 onwards. Response was determined by the investigator using RECIST v1.1. Objective response rate was determined in patients with measurable disease at baseline, and was defined as a complete or partial response on two consecutive occasions ≥ 4 weeks apart. Disease control rate was defined as the proportion of patients achieving a complete response, partial response, or stable disease.

### Statistical analysis

Traditional 3 + 3 design was adopted in this study and so sample size calculation based on statistical evaluation was not performed. The planned sample size was three-to-six patients. All analyses were performed on the safety-evaluable population, which comprised all patients who received any amount of study drug. Data are presented at the time of study completion (last patient last visit: February 28, 2020). Dose intensity was defined as the actual number of doses/planned number of doses × 100. The planned number of doses was 1 + (last dose date—first dose date)/21. Therefore, the effect of dose delay is reflected in dose intensity.

## Results

### Patients

Between November 2019 and January 2020, three patients were enrolled with diagnoses of NSCLC, pancreatic cancer, and cholangiocarcinoma (Table 1). Median patient age was 69.3 years (range 64.0–75.0), and all patients had an ECOG PS of 0 at screening. All patients had received at least one prior chemotherapy regimen in the metastatic setting, and one patient had received prior anti-PD-L1/PD-1 therapy. All patients were evaluable for safety, tolerability, PK, and anti-tumor assessments. At the time of last patient last visit, all three patients had discontinued study treatment due to PD.

**Table 1:** Baseline demographic and clinical characteristics

Characteristic	Tiragolumab + atezolizumab (*n* = 3)
Median age, years (range)	69.3 (64.0–75.0)
Male, *n* (%)	1 (33.3)
ECOG PS 0, *n* (%)	3 (100.0)
Diagnosis, *n* (%)	
NSCLC	1 (33.3)
Pancreatic	1 (33.3)
Cholangiocarcinoma	1 (33.3)
Primary tumor, *n* (%)	
Yes	2 (66.7)
No	1 (33.3)
Prior cancer therapy, *n* (%)	
Radiotherapy	0.0
Chemotherapy^a^	3 (100.0)
Surgery	2 (66.7)
Immunotherapy^b^	1 (33.3)

*ECOG PS* Eastern Cooperative Oncology Group performance status, *NSCLC* non-small cell lung cancer

^a^Includes adjuvant and metastatic settings

^b^Patient received prior anti-programmed death-1/programmed death-ligand 1 therapy, nivolumab

### Safety

Patients received a mean (standard deviation [SD]) of 3.3 (1.2) cycles of combination therapy, with a mean (SD) dose intensity of 98.1% (3.2%; Table 2). No DLTs occurred during the DLT assessment window. Nine AEs were recorded in the three patients, at a maximum intensity of grade 2. Treatment-related AEs occurred in two patients (Table 3) and included stomatitis, pneumonia, aspartate aminotransferase increased, arthralgia, pruritus, and rash. There were no incidences of infusion-related reactions. There were no grade ≥ 3 AEs, serious AEs, AEs leading to discontinuation of either study drug, AEs leading to dose modification or withdrawal of any study drug, or AEs leading to death. No clinically significant laboratory abnormalities or changes in vital signs were observed. Electrocardiographic abnormalities were seen in two patients during treatment, but these were not deemed clinically significant. All three patients were negative for ADAs to tiragolumab and atezolizumab at baseline and following study treatment.

**Table 2:** Study drug exposure (safety population)

Characteristic	Tiragolumab + atezolizumab (*n* = 3)	
Observation duration, days		
Mean (SD)	75.3 (22.1)	
Median	85.0	
Min–max	50.0–91.0	
Treatment duration^a^, days		
Mean (SD)	51.7 (25.8)	
Median	64.0	
Min–max	22.0–69.0	
Number of cycles, *n*		
Mean (SD)	3.3 (1.2)	
Median	4.0	
Min–max	2.0–4.0	
Dose intensity^b^, %		
Mean (SD)	98.1 (3.2)	
Median	100.0	
Min–max	94.4–100.0	
Cumulative dose, mg	Tiragolumab	Atezolizumab
Mean (SD)	2000.0 (692.8)	4000.0 (1385.6)
Median	2400.0	4800.0
Min–max	1200.0–2400.0	2400.0–4800.0

*SD* standard deviation

^a^Defined as the date of the last dose of study drug, minus the date of the first dose, plus 1 day

^b^Defined as a proportion of infusions received to planned infusions

**Table 3:** Treatment-related adverse event summary

Patients with events, *n* (%) System organ class/preferred term	Tiragolumab + atezolizumab (*n* = 3)	
	Any grade	Grade ≥ 3
Any treatment-related AE	2 (66.7)	0.0
Gastrointestinal disorders	1 (33.3)	0.0
Stomatitis	1 (33.3)	0.0
Infections and infestations	1 (33.3)	0.0
Pneumonia	1 (33.3)	0.0
Investigations	1 (33.3)	0.0
AST increased	1 (33.3)	0.0
Musculoskeletal and connective tissue disorders	1 (33.3)	0.0
Arthralgia	1 (33.3)	0.0
Skin and subcutaneous tissue disorders	2 (66.7)	0.0
Pruritus	1 (33.3)	0.0
Rash	1 (33.3)	0.0

*AE* adverse event, *AST* aspartate aminotransferase

Multiple occurrences of the same AE in one individual are counted once at the greatest intensity for that preferred term

### Pharmacokinetics

**Fig. 1:**
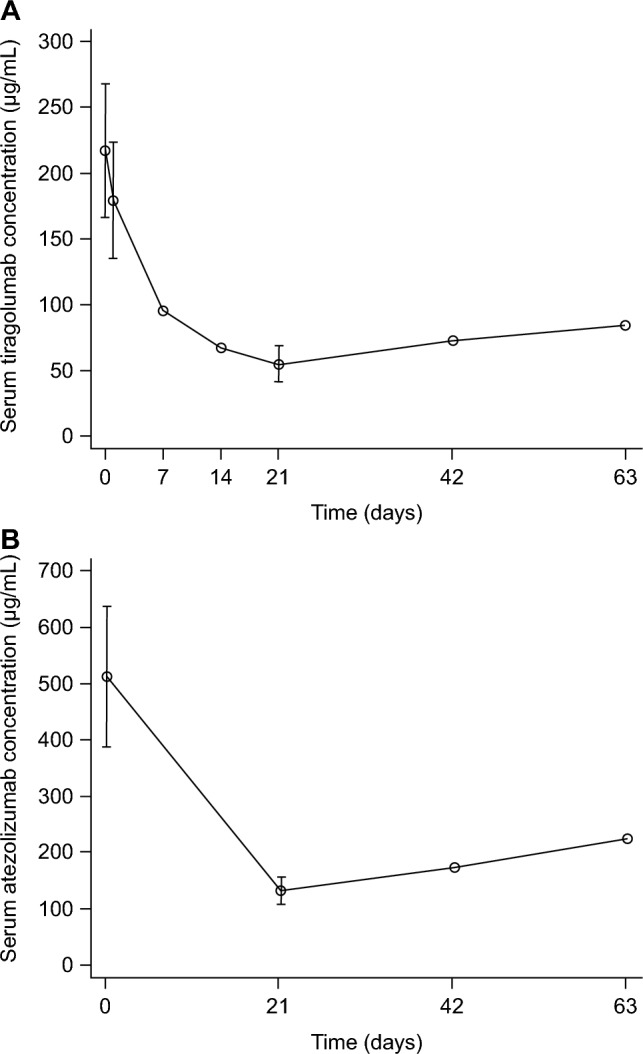
Mean (± SD) serum concentration–time profiles for **a** tiragolumab and **b** atezolizumab following single-dose administration. Mean C_min_ of atezolizumab was 174 μg/mL in cycle 3 and 225 μg/mL in cycle 4. Time 0 means cycle 1 day 1. The same applies to the other x-axis numbers. Blood samples were unable to be collected on day 1 of cycle 8. **a** The circle between Time 0 and 7 is the value of Time 1 (cycle 1, day 2). Days 7, 14, 43, and 64 of cycle 1 have no error bars, because only two samples were measured. *C*_*min*_ maximum serum concentration, *SD* standard deviation

**Fig. 2:**
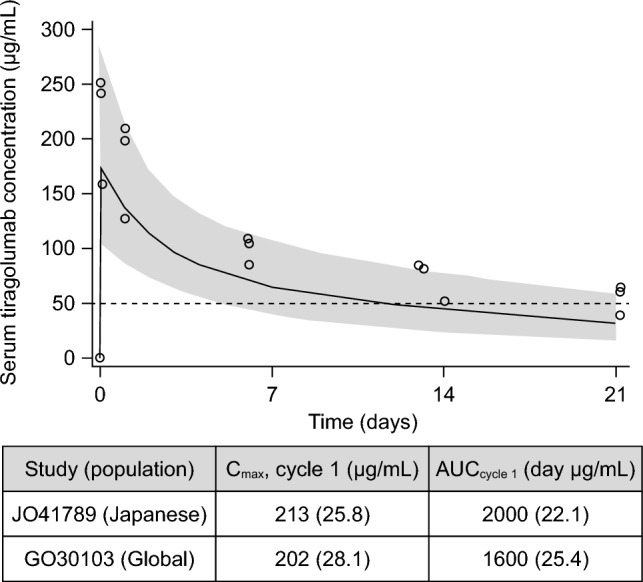
Median (90% prediction interval) serum concentration of tiragolumab during cycle 1. Open circles represent observed data. The solid black line represents the fitted mean with 90% CI (shaded area). The area under the concentration–time curve from 0 to the last measurable concentration was 2000 ± 405 μg·day/mL and elimination half-life was 17.6 ± 3.72 days. *AUC*_*cycle 1*_ area under the concentration–time curve in cycle 1, *CI* confidence interval, *C*_*max*_ maximum serum concentration

The C_max_ was collected 30 min after the end of infusion treatment. In cycle 1, mean (± SD) PK parameters of tiragolumab were as follows: C_max_ 217 ± 50.7 μg/mL (Fig. 1a), C_min_ 54.9 ± 13.9 μg/mL, AUC_last_ 2000 ± 405 μg·day/mL, and t_1/2_ 17.6 ± 3.72 days. Mean pre-dose serum concentration of tiragolumab remained consistent after cycle 3. In cycle 1, mean (± SD) C_max_ of atezolizumab was 512 ± 124 μg/mL (Fig. 1b). Mean pre-dose serum concentration of atezolizumab was 174 μg/mL in cycle 3 and 225 μg/mL in cycle 4. Using a population PK model used at the time of consultation with the authorities for Japan's participation in a global phase III study, median and 90% prediction interval of serum tiragolumab concentration were calculated during cycle 1. No differences in tiragolumab PK characteristics were noted between Japanese (C_max_ 213 μg/mL, AUC_cycle1_ 2000 μg·day/mL) and non-Japanese patients (C_max_ 202 μg/mL, AUC_cycle1_ 1600 μg·day/mL; Fig. 2).

### Anti-tumor activity

Best overall response was stable disease in two patients. The first patient with stable disease was female, aged 75 with NSCLC and heavily pre-treated with > 5 lines of prior therapy (including anti-PD-L1/PD-1 therapy). This patient received four cycles of treatment and their PFS was 1.8 months. The second patient with stable disease was male, aged 69 with cholangiocarcinoma and had received three prior lines of therapy. This patient received four cycles of treatment, and their PFS was 2.8 months. The third patient was female, aged 64 with pancreatic cancer and had received three prior lines of therapy. Best response in this patient was stable disease of the target lesion on day 42, but their disease progressed, and best overall response could not be determined (PFS 1.4 months, censored).

## Discussion

A number of anti-TIGIT monoclonal antibodies are currently being investigated in clinical trials, either as monotherapy or in combination with anti-PD-L1/PD-1 antibodies for the treatment of advanced or metastatic solid tumors [9, 11, 14, 15]. In this open-label phase I study, we found that the anti-TIGIT monoclonal antibody tiragolumab in combination with the anti-PD-L1 monoclonal antibody atezolizumab was well tolerated in a small sample of Japanese patients with advanced or metastatic solid tumors. No DLTs occurred, and there were no grade ≥ 3 AEs, serious AEs, AEs leading to discontinuation of either study drug, AEs leading to dose modification or withdrawal of any study drug, or AEs leading to death. The AE profile was similar to that reported in the larger global phase Ia/Ib GO30103 study [13], and no new safety signals were identified. Tiragolumab reached C_max_ at approximately 30 min after completion of treatment and was expected to reach steady-state levels by cycle 1. The mean serum drug concentration of atezolizumab after cycle 3 was similar to the previously reported mean serum drug concentration in Japanese patients following multiple-dose administration of single-agent atezolizumab (162 μg/mL before cycle 3 and 188 μg/mL before cycle 4 [Chugai data on file]). Thus, there was no major effect on the PK of atezolizumab when given in combination with tiragolumab. Importantly, no differences were noted in tiragolumab PK characteristics between Japanese patients enrolled in this study and non-Japanese patients enrolled in the global phase Ia/Ib GO30103 study, although PK values were not directly compared [13].

Best overall response was stable disease in two patients. Both patients with stable disease were heavily pre-treated (≥ 4 prior lines of treatment); one of these patients had received prior treatment with nivolumab and may have developed resistance to anti-PD-L1/PD-1 therapy. However, promising anti-tumor activity was reported with the combination of tiragolumab and atezolizumab in the GO30103 study, with a confirmed objective response rate of 46% in the subset of patients with PD-L1-positive NSCLC [13].

Limitations of the study include that the study did not include a traditional dose-escalation process, and the dose–response relationship of tiragolumab was not investigated in this patient population. When reporting t_1/2_, it is difficult to calculate the exact half-life based on PK data obtained within a 21-day dosing interval; therefore, the current data may not be adequate. In addition, the small sample size of the study (*N* = 3) makes it difficult to draw meaningful conclusions, and caution is required when interpreting secondary tumor response results. The results of this phase 1 study are also limited by the small sample size for different tumor types and by the poor prognosis of the study population, with all three patients having received prior therapy. Further validation of these results is needed from additional phase II and III studies.

## Conclusion

Tiragolumab in combination with atezolizumab was well tolerated in this small sample of Japanese patients with advanced or metastatic solid tumors. No differences in tiragolumab PK characteristics were noted between Japanese patients in this study when compared with non-Japanese patients in the GO30103 study; however, PK values were not directly compared. These data may support the inclusion of Japanese patients in ongoing pivotal global phase III studies of tiragolumab, including: SKYSCRAPER-01 (NCT04294810) in patients with advanced PD-L1-positive NSCLC, SKYSCRAPER-03 (NCT04513925) in patients with locally advanced, unresectable stage III NSCLC, SKYSCRAPER-06 (NCT04619797) in patients with previously untreated advanced non-squamous NSCLC, and SKYSCRAPER-07 (NCT04543617) in patients with unresectable esophageal squamous cell carcinoma.

### Supplementary Information

Below is the link to the electronic supplementary material.

Supplementary file1 (DOCX 18 KB)

## Data Availability

Qualified researchers may request access to individual patient-level data through the clinical study data request platform (www.clinicalstudydatarequest.com). For further details on Chugai's Data Sharing Policy and how to request access to related clinical study documents, see here (www.chugai-pharm.co.jp/english/profile/rd/ctds_request.html).
