# Correction to: Phase I study of the anti‑TIGIT antibody tiragolumab in combination with atezolizumab in Japanese patients with advanced or metastatic solid tumors

**DOI:** 10.1007/s00280-024-04645-9

**Published:** 2024-03-31

**Authors:** Noboru Yamamoto, Takafumi Koyama, Jun Sato, Tatsuya Yoshida, Kazuki Sudo, Satoru Iwasa, Shunsuke Kondo, Kan Yonemori, Atsuko Kawasaki, Kyoko Satake, Shoyo Shibata, Toshio Shimizu

**Affiliations:** 1 Department of Experimental Therapeutics, National Cancer Center Hospital, Tsukiji 5-1-1, Chuo-Ku, Tokyo, 104–0045 Japan; 2 Chugai Pharmaceutical Co., Ltd, 1-1 Nihonbashi-Muromachi 2-Chome Chuo-Ku, Tokyo, 103–8324 Japan

**Correction to: Cancer Chemotherapy and Pharmacology** 10.1007/s00280-02-04627-3.

In the original publication, the authors have identified a minor error within the abstract of conclusion section.

The incorrect text appeared as follows:

Tiragolumab plus atezolizumab was well tolerated in Japanese patients with advanced/metastatic solid tumors, and no differences in tiragolumab PK characteristics were noted between Japanese patients enrolled in this study, and non-Japanese patients enrolled in a global phase III study.

The text in the conclusion section should now read as below:

Tiragolumab plus atezolizumab was well tolerated in Japanesepatients with advanced/metastatic solid tumors, and no differences in tiragolumab PK characteristics were noted between Japanese patients enrolled in this study, and non-Japanese patients enrolled in a global phase Ia/Ib study.

The original article has been corrected.

