# Supplementary material for: Phase I study of the anti-TIGIT antibody tiragolumab in combination with atezolizumab in Japanese patients with advanced or metastatic solid tumors
Source: Cancer Chemother Pharmacol. 2024 Jan 11;94(1):109–15. doi: 10.1007/s00280-023-04627-3 (PMC11258096; doi:10.1007/s00280-023-04627-3)
Supplement: Supplementary file 1 — Supplementary file1 (DOCX 18 KB) [file 280_2023_4627_MOESM1_ESM.docx]

**Supplementary materials**

**Definition of dose-limiting toxicities (DLTs)**

DLTs were defined as any one of the following adverse events considered by the investigator to be related to tiragolumab or atezolizumab occurring during the DLT assessment window:

- Grade ≥ 3 non-hematologic, non-hepatic adverse event, with the following exceptions:
  - grade 3 nausea, vomiting, or diarrhea that resolves to grade ≤ 2 within 3 days (with/without treatment)
  - grade 3 fatigue that resolves to grade ≤ 2 within 3 days
  - grade 3 fever (> 40°C with recovery within 24 hours)
  - grade 3 tumor flare (defined as local pain, irritation, or rash at sites of known or suspected tumor) that resolves to grade ≤ 2 within 7 days
  - grade ≥ 3 laboratory abnormalities that are asymptomatic and considered by the investigator to be clinically insignificant, and that resolve to grade ≤ 2 within 7 days
  - grade 3 rash that resolves to grade ≤ 2 within 7 days with therapy equivalent to prednisone 10 mg/day or less
- Grade 4 neutrophil count decreased beyond 7 days or requiring granulocyte-colony stimulating factor therapy
- Febrile neutropenia
- Grade 4 anemia, or anemia requiring blood transfusions
- Grade 4 platelet count decreased, or grade 3 platelet count decreased requiring platelet transfusions
- Alanine aminotransferase or aspartate transaminase increased > 3 x upper limit of normal, and total bilirubin increased > 2 x upper limit of normal
- Events requiring treatment interruption for > 14 days.

**DLT tolerability evaluation criteria**

Based on the incidence of DLTs observed during the DLT evaluation period, the tolerability of

tiragolumab + atezolizumab combination therapy was evaluated according to the following criteria

listed below. When assessing tolerability, the occurrence of adverse events was also considered for

all patients enrolled in the Cohort, including those who were not eligible for DLT assessment.

- If no DLTs were present in one of three patients evaluable for DLT, it was considered tolerable.
- If DLTs were present in one of three patients evaluable for DLT, three additional patients were enrolled in the Cohort. It was considered tolerable if DLT occurred in one of six patients evaluable for DLT (if three DLT evaluable patients did not develop DLT).
- If DLT was present in at least two patients evaluable for DLT, a new patient was not enrolled into the Cohort.
